# The effects of stimulating the cerebellum on social sequences: A tDCS-fMRI pilot study: Los efectos de estimular el cerebelo en secuencias sociales: Un estudio piloto con tDCS y fMRI

**DOI:** 10.1016/j.ijchp.2023.100373

**Published:** 2023-01-28

**Authors:** Beatriz Catoira, Frank Van Overwalle, Peter Van Schuerbeek, Hubert Raeymaekers, Elien Heleven, Kris Baetens, Natacha Deroost, Chris Baeken

**Affiliations:** a Department of Psychiatry (UZ Brussel), Vrije Universiteit Brussel, Brussels, Belgium; b Ghent Experimental Psychiatry (GHEP) Lab, Ghent University, Corneel Heymanslaan 10, Ghent 9000, Belgium; c Department of Psychology and Center for Neuroscience, Vrije Universiteit Brussel, Brussels, Belgium; d Department of Radiology, UZ Brussel, Brussels, Belgium; e Department of Head and Skin, Psychiatry and Medical Psychology, Ghent University Hospital, Ghent, Belgium; f Department of Electrical Engineering, Eindhoven University of Technology, the Netherlands

**Keywords:** Cerebellum, tDCS, fMRI, Mentalizing, Picture sequencing

## Abstract

Research on the involvement of the cerebellum in social behavior and its relationship with social mentalizing has just begun. Social mentalizing is the ability to attribute mental states such as desires, intentions, and beliefs to others. This ability involves the use of social action sequences which are believed to be stored in the cerebellum. In order to better understand the neurobiology of social mentalizing, we applied cerebellar transcranial direct current stimulation (tDCS) on 23 healthy participants in the MRI scanner, immediately followed by measuring their brain activity during a task that required to generate the correct sequence of social actions involving false (i.e., outdated) and true beliefs, social routines and non-social (control) events. The results revealed that stimulation decreased task performance along with decreased brain activation in mentalizing areas, including the temporoparietal junction and the precuneus. This decrease was strongest for true belief sequences compared to the other sequences. These findings support the functional impact of the cerebellum on the mentalizing network and belief mentalizing, contributing to the understanding of the role of the cerebellum in social sequences.

## Introduction

The cerebellum is traditionally known for its involvement in motion. One of its major functions is to store internal models or representations of body part dynamics, key to executing and fine-tuning movements (Ito, 2008). For example, when one drinks a glass of water, the wrist tilts the glass while the lips approach it. To prevent water overflow, the brain applies a model that predicts when the water will reach your mouth. According to the “Sequence Detection'' hypothesis (Leggio & Molinari, 2015), the cerebellum detects and reconstructs patterns of events structured in time and space. The reconstruction of these sequences is the core of the internal models that are used to predict upcoming events.

More recently, it has become clear that the cerebellum also contains non-motor representations of real and imaginary situations in internal models, used to understand current events and to anticipate future events (Ito, 2008). Accordingly, the cerebellum also stores internal models of social interactions (for example, how to greet someone with a handshake, or how to have a fluent conversation without interrupting each other), which explains the involvement of the cerebellum in cognitive and social processes (Ito, 2008; Van Overwalle et al., 2014; Van Overwalle & Mariën, 2016). On the neuroanatomical level, a distinction is made between the anterior part of the cerebellum (important for the coordination of voluntary movements), and the posterior part of the cerebellum (involved in cognitive, social, and affective processes) (Van Overwalle et al., 2014).

Particularly, the lateral hemispheres of the posterior cerebellum have been associated with complex cognitive social processes such as mentalizing (Van Overwalle et al., 2014; Leggio & Olivito, 2018). Mentalizing is the cognitive ability to attribute mental states, such as desires, intentions, and beliefs, to other people (Frith & Frith, 1999) and it is fundamental for understanding and predicting other people's behavior. False belief stories are often used to measure mentalizing abilities (Wimmer & Perner, 1983). In false belief stories, a character in the story is not aware of something that happens (e.g., a toy is displaced during a child's absence) and acts upon false beliefs that are logical from that character's perspective (e.g., the child will search for the toy at its original location; Wimmer & Perner, 1983). However, these false beliefs are not in line with the real situation, and therefore they will differ from the beliefs of an observer (a participant who performs the task and knows the real location of the toy). False belief sequences have shown differential activation in the right posterior cerebellar Crus area when compared to mechanical sequences (Heleven et al., 2019).

Problems with mentalizing, including the attribution of false beliefs to others, are present in several disorders such as autism spectrum disorder (ASD) (Heleven, Bylemans, Ma, Baeken, & Baetens, 2022) or schizophrenia (Hyatt et al., 2020). Patients with cerebellar damage have been shown to perform worse than neurotypical controls on tasks that depict sequences of social actions, particularly when those pictures depict biological human actions (Cattaneo et al., 2012) or social sequences that include false beliefs (Van Overwalle et al., 2019a).

A network that has been associated with mentalizing processes is the default mode network (DMN) (Li et al., 2014) or mentalizing network (Van Overwalle, 2014). The DMN is defined as a set of brain areas that are activated when humans are not focused on their external environment (Buckner et al., 2008). During mentalizing, the bilateral temporo-parietal junction (TPJ) (Samson et al., 2004) is thought to be important for inferring the temporary mental states of other people, and the medial prefrontal cortex (mPFC) for inferring more enduring social information (e.g., personality traits) of the self and others (Van Overwalle, 2009). In addition, the cerebellum has been shown to be connected to the DMN in resting state (Buckner et al., 2011), task-based meta-analysis (Van Overwalle & Mariën, 2016), and dynamic causal modeling fMRI analysis (Van Overwalle et al., 2020, 2019b).

A way to influence the mentalizing network is with the use of transcranial direct current stimulation (tDCS). Modulation of mentalizing processes using tDCS has mostly targeted regions such as the TPJ (Salehinejad et al., 2021) or the mPFC (Cotelli et al., 2020). Due to the high density of neurons in the cerebellum and multisynaptic connections to the cerebrum (Galea et al., 2009), it is more difficult to predict the effects of cerebellar as compared to cerebral tDCS (van Dun et al., 2016; Oldrati & Schutter, 2018). Nonetheless, a recent study using cerebellar tDCS showed that anodal tDCS increased performance on a mentalizing task (Clausi et al., 2022). Another study (Rice et al., 2021) targeted two cerebellar regions (anterior sensorimotor versus cognitive posterolateral) and showed differential effects (the language network was only affected with posterior cerebellar stimulation). In addition, a study targeting the posterior cerebellum with low-frequency repetitive transcranial magnetic stimulation (Heleven et al., 2021) increased performance in a picture sequencing task involving social beliefs, supporting the causal role of the cerebellum in the generation of mentalizing sequences. Although this pioneering work is promising, there is still a lack of sufficient non-invasive brain stimulation studies that target the involvement of the cerebellum in mentalizing action sequences.

In the present study, we aimed to explore the effects of cerebellar tDCS using a picture sequencing task with social beliefs in the MRI environment. We combined tDCS and fMRI to assess the effects of anodal stimulation at the right posterior cerebellum over the mentalizing network. Given earlier stimulation studies reviewed above, we expected to increase task performance and activation in social mentalizing areas of the cerebellum and cerebrum after anodal tDCS, especially for pictures requiring mentalizing (i.e., true and false beliefs).

## Methods

### Participants

A total of 23 healthy right-handed participants (ages 18- 35; 17 females and 6 males) were included. Due to technical issues, only 19 participants are included in the fMRI analysis. Participants were recruited posting flyers on social media. To assess eligibility, participants completed digitally a screening form for tDCS and fMRI, a modified version of the MINI screen (Mini International Neuropsychiatric Interview, version 5.0.0; Lecrubier et al., 1998; Dutch version by Overbeek et al., 1999), the Beck Depression Inventory (BDI-II; Beck et al., 1996; Dutch version by van der Does, 2002) and the Autism Spectrum Quotient (AQ, Baron-Cohen et al., 2001; Dutch version by Hoekstra et al., 2008) questionnaires. Participants with current psychiatric disorders or medication, a score >14 on the BDI or >32 in the AQ were excluded.

### Study design

This study consisted of a within-subject, counterbalanced sham-controlled design. Each participant performed two visits with identical protocol except for the type of tDCS applied (anodal or sham). The interval between sessions varied between 24 h and 2 weeks due to time constraints.

**Fig. 1:**
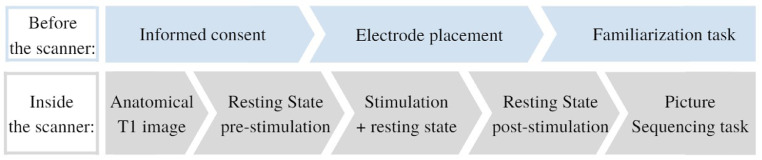
Scheme of the different parts of the session.

Participants signed the informed consent before the start of each session. They could ask questions and were explained the procedures of the session (see Fig. 1). Next, the tDCS electrodes were positioned. Then they performed a familiarization task on a computer outside of the scanner. Subsequently, the participant was placed inside the scanner. While in resting state, tDCS was administered. Next, the picture sequencing task was performed in the scanner. After study completion, monetary compensation (80 euros) was provided. The study was approved by the Medical Ethics Committee of the Vrije Universiteit Brussel (Universitair Ziekenhuis Brussel).

### tDCS protocol

An MRI-compatible tDCS device (model 1300A Soterix Medical, New York, NY, USA) was used. The current ramped up for 30 s, then the intensity was 2 mA for 20 min and then it ramped down for 30 s. The sham condition had a ramp-up and down to mimic the skin sensations but had no real stimulation during the 20 min in between. tDCS was applied via two rubber electrodes (sizes 4.5 × 4.5 or 5 × 5 cm; distinct sizes were used due to technical breakdown and unavailability of replacement) with Ten20 Paste gel (Weaver) and fixed with rubber bands.

Based on SimNibs simulations (version 3.2.1; Thielscher et al., 2015) we placed the anode over PO10 position (EEG 10–20 system) and the cathode over Iz in order to maximize the focality over the right posterior cerebellum (MNI coordinates *X* = −25, *Y* = −75, *Z* = −40).

### fMRI acquisition

Data were acquired on a 3T Discovery MR750w scanner using a 24-channel head coil (GE Medical Systems, Milwaukee, WI, USA) at the UZ Brussel (Belgium). First, we obtained a T1-weighted anatomical scan (3D BRAVO, 432 sagittal slices, 0.8 mm slice thickness, FoV 25.6 × 25.6 mm, TR = 8.2 ms, TE = 3.2 ms, TI = 450 ms, Flip angle = 12º). In a following step, approximately 8 min after the stimulation ended, gradient multi-echo functional scans (57 slices per location, slice thickness of 2.5 mm, FoV 22.0 × 22.0 mm, 2 echoes: TE1 = 22.5 TE2= 62.6 ms, TR = 2000 ms, Flip angle = 52º) were collected during the picture sequencing task.

### Task

The picture sequencing task was built using E-Prime (Psychology Software Tools, Sharpsburg, PA, USA) and it was displayed in the scanner using a mirror. This task was originally developed by Langdon and Coltheart (1999) and extended by Heleven et al. (2019).

To familiarize participants with the stimuli beforehand, participants performed a familiarization task with a set of four pictures (cartoon-like drawings) that depicted a sequence of events. Participants had to answer yes or no questions about these events.

**Fig. 2:**
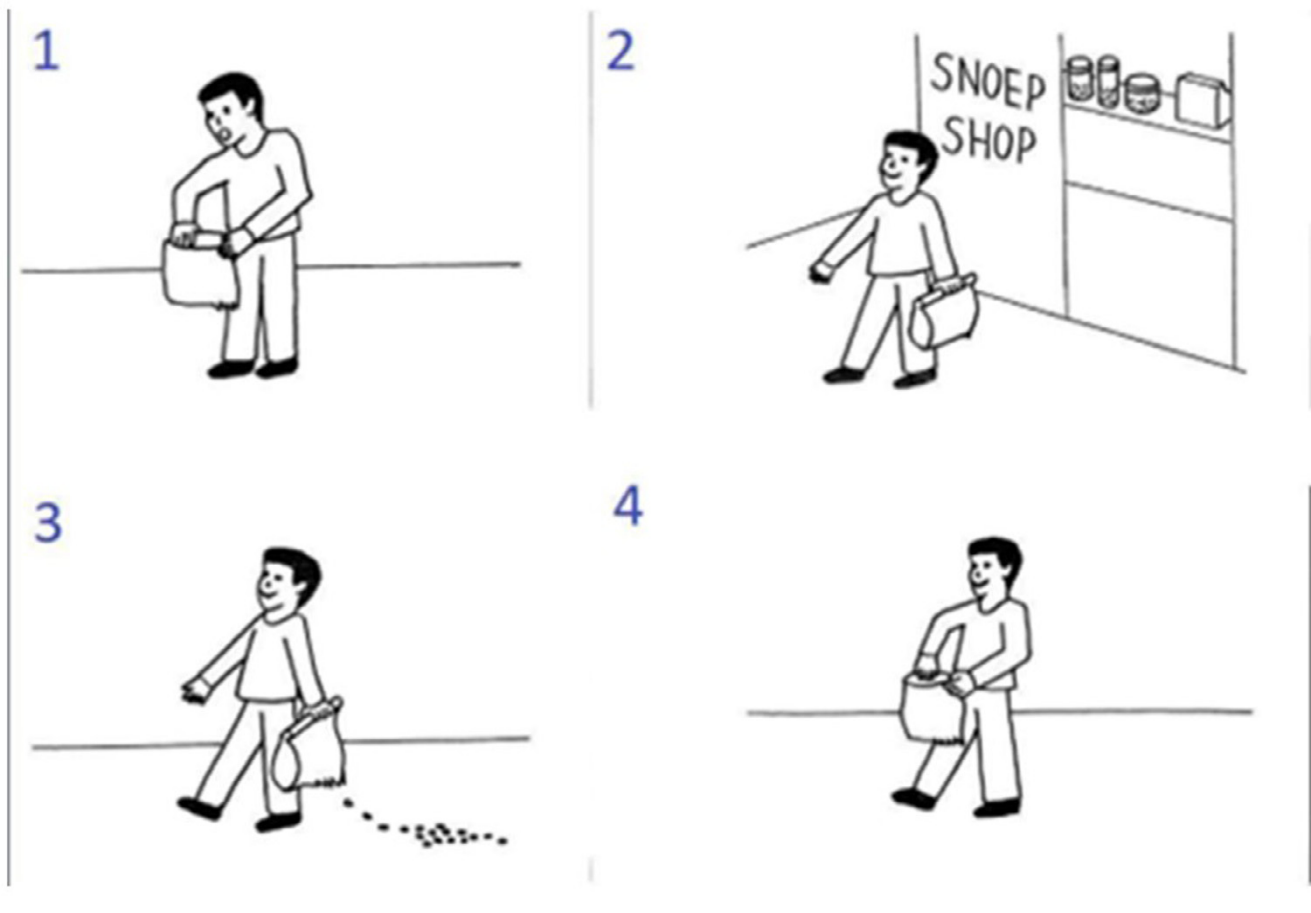
Example of a false belief sequence from the picture sequencing task. This sequence is presented in a scrambled order. The correct order is 2: the central character leaves the shop; 3: while the central character is unaware, the candy falls out of the bag; 4: the central character is going to open the bag, still smiling because he has the false belief that the candy will still in the bag; 1: the central character acts surprised because he is not expecting the candy to be gone.

The experimental trials consisted of a set of four pictures that illustrated a sequence in scrambled order (an example is shown in Fig. 2). For the experimental trials, the goal of the participant was to select the pictures in the right order via button press (first, second and third button press would select the first, second, and third image; and the fourth button press would be used to confirm the selected order or to abort the trial and restart the sequence). There were two practice trials and then sixteen experimental trials (4 per condition) per session, in randomized order.

This task has four different conditions: false beliefs, true beliefs, social scripts and mechanical events. In Fig. 2 we can see an example of a false belief sequence. In this condition, the central character holds a false belief because they are unaware of a change in the environment (in Fig. 2, the main character is unaware that the candies have fallen) and therefore they act surprised or angry. To select the correct order, the participant must be aware that they, as an observer, have more information than the central character of the sequence and must acknowledge the mismatch in information. True beliefs are similar to false beliefs, but the central character does not hold any false belief. Social scripts function as a social non-mentalizing control and involve well-known routine action sequences (like brushing teeth or washing hands). Mechanical events serve as a non-social control which depicts purely action-reaction sequences that mainly involve objects. After each trial participants were asked a confidence question (How sure are you of your answer?) and rated it from 1 (not at all) to 4 (very much).

### Behavioral analysis

We measured accuracy following the scoring system of the original task (Langdon & Coltheart, 1999). According to this system, a correct answer for the first and last picture gives 2 points each, while the correct second and third picture score 1 point each. This amounts to a maximum of 6 points. We also analyzed the reaction time from the stimuli presentation until the first button press. The logic behind this measurement is that it assumes that participants first perform the critical sequencing mental calculations and then start executing the motor answers. The two outcomes were analyzed separately using a Mixed Model ANOVA in SPSS (IBM Corp. Released 2019. IBM SPSS Statistics for Windows, Version 26.0. Armonk, NY: IBM Corp). As suggested in previous studies (Heleven et al., 2019; Heleven, Bylemans, Ma, Baeken, & Baetens, 2022), we added the AQ scores as a covariate for the analysis.

### fMRI processing and data analysis

Neuroimaging data was preprocessed and analyzed using SPM 12 (Wellcome Department of Cognitive neurology, London, UK); extra SPM-based toolboxes were used for some steps of the preprocessing.

The first step was to convert the raw images into .nii files using MRIcro GL (Rorden & Brett, 2000; https://www.nitrc.org/projects/mricrogl). All images were reoriented to the AC-PC plane. Then the 4D to 3D file conversion of SPM12 was used, after that the two echoes were merged using the Multi-Echo and Hyperband toolbox (MEHB fMRI; https://github.com/P-VS/MEHBfMRI). The ACID toolbox (http://diffusiontools.com/) was used to perform the susceptibility correction using a phase polarity inverted scan. The next step was to perform slice time correction from the MEHB toolbox. Then, functional images were coregistered to the anatomical T1 image. Afterwards, the images were normalized to MNI-152 template and smoothed with a Gaussian Kernel (8 mm FWHM). Finally we examined the data for excessive motion using the Artifact Detection Tool (ART; http://web.mit.edu/swg/art/art.pdf; http://www.nitrc.org/projects/artifact_detect). A single participant had to be excluded from further analysis due to excessive motion (z-threshold > 3.0; movement threshold > 0.5). The six directions of the motion parameters were included as nuisance regressors.

For the single participant (first) level of analysis, we modeled an event-related design for each Picture condition (false belief, true belief, social script and mechanical) with the presentation of each sequence as onset. Following Heleven et al. (2019), the duration was set to 4 s, reflecting the expected minimal time across all sequences to perform the critical sequencing mental calculations.

At the group (second) level we chose a whole brain within-participants one-way ANOVA to contrast Stimulation (real versus sham) across Picture conditions. Since we had a priori hypothesis about which areas would be affected by the stimulation, we chose several regions of interest (ROI) from the networks previously involved in the picture sequencing task (mentalizing, goal-directed action observation and action sequencing), using a small volume correction (i.e., correcting for multiple comparisons in the ROI instead of the whole brain). The coordinates were extracted from meta-analyses on the cortex (Van Overwalle, 2009; Van Overwalle & Baetens, 2009) and the cerebellum (Van Overwalle et al., 2020). The ROIs involved a sphere with radius of 10 mm and were centered around the MNI coordinates of the cerebellar crus II (*x* = ±25, *y* = −75, *z* = −40), cerebellar crus I (*x* = ±40, *y* = −70, *z* = −40), mPFC (*x* = 0, *y* = 50, *z* = 20), TPJ (*x* = ±50, *y* = −55, *z* = 25), precuneus (*x* = 0, *y* = −60, *z* = 40), premotor cortex (*x* = ±40, *y* = 5, *z* = 40), anterior inferior parietal sulcus (a-IPS; *x* = ±40, *y* = −40, *z* = 45) and posterior superior temporal sulcus (PSTS; *x* = ±50, *y* = −55, *z* = 10).

To expand our findings to regions besides the expected networks, we examined a whole-brain analysis of the same contrasts. We used a voxel-wise threshold of *p* 〈 0.005 and a minimal cluster size of k 〉 10 to identify clusters of activation, and to correct for multiple comparisons we used random field theory-based Family-Wise Error rate (Chumbley et al., 2010) with a significance of *p* < 0.05 on each cluster provided by the xjView toolbox (https://www.alivelearn.net/xjview), to identify significant clusters.

## Results

### Behavioral results

For our two measures we performed a mixed model ANOVA using as within participant factors: Picture (false belief, true belief, social script or mechanical), Stimulation (anodal or sham), and Session (1 or 2). Results are listed in Table 1.

**Table 1:** *Means and standard deviations of the Picture Sequencing task*.

		false belief		true belief		social script		mechanical	
		mean	SD	mean	SD	mean	SD	mean	SD
**Accuracy**									
Session 1	Stim	2.44	2.26	3.00	2.52	3.48	2.50	3.33	2.32
	Sham	3.29	2.57	4.13	2.22	5.09	1.47	4.22	2.21
Session 2	Stim	3.14	2.59	3.52	2.62	4.41	2.13	4.36	2.34
	Sham	2.86	2.52	3.29	2.11	5.38	1.19	4.08	2.52
**RT (seconds)**									
Session 1	Stim.	33.27	13.23	27.81	7.98	26.52	9.72	24.73	8.56
	Sham	27.69	11.13	26.01	11.69	24.33	9.92	23.10	7.76
Session 2	Stim	26.52	10.45	28.99	12.05	22.26	12.11	20.69	8.71
	Sham	29.36	10.28	30.83	11.02	23.07	8.91	24.54	7.76

Note: Accuracy ranged from 0 to 6 points according to the original scoring schema by Langdon and Coltheart (1999). RT: reaction time, measured in seconds; SD: standard deviation; Stim: tDCS stimulation.

### Accuracy

For the accuracy measure, the main effect of Picture was significant (*F* = 7.411, *p* < 0.001). Pairwise comparisons revealed lower accuracy for false beliefs than social scripts (*p* < 0.001) and mechanical stories (*p* < 0.01), and for true beliefs than social scripts (*p* < 0.01). There was a significant interaction between Stimulation and Session (*F* = 4.160, *p <* 0.05), showing that participants were less accurate overall (i.e., for all stories) after stimulation compared to sham during the first session, but not during the second session.

### Response time

**Fig. 3:**
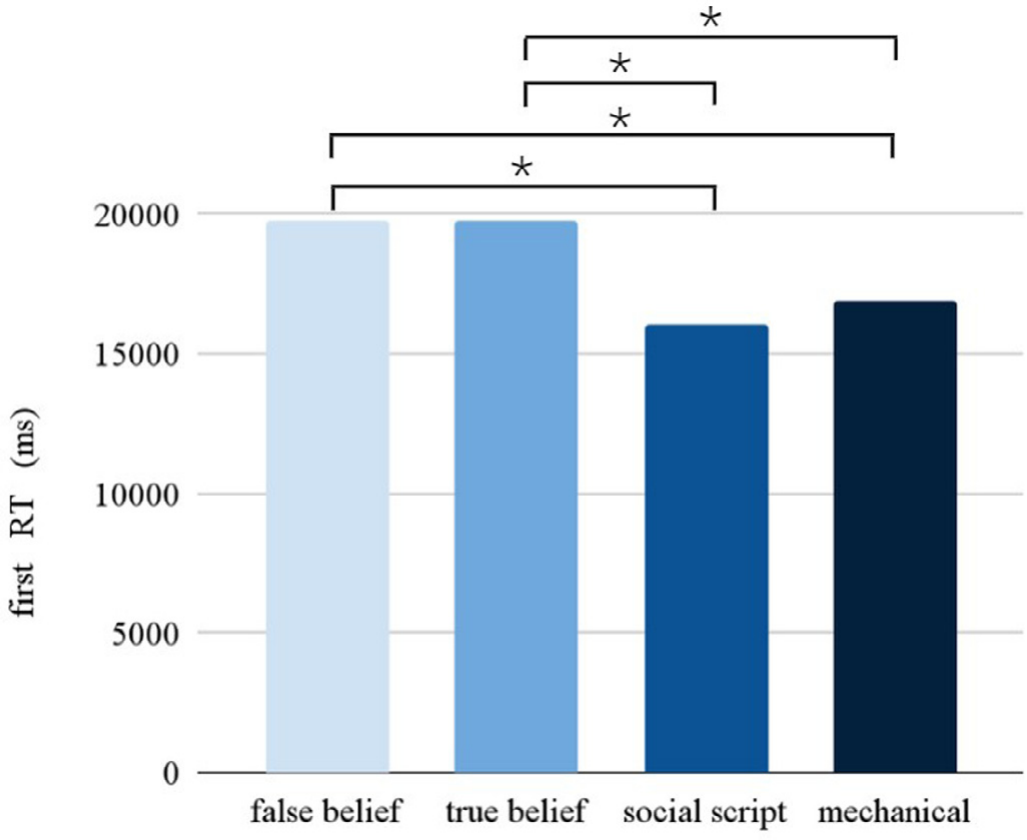
Pairwise comparisons between the different Picture conditions (*p* < 0.005). Participants required more time to respond to the (more complex) false and true belief stories, while they required less time for the social script and mechanical stories.

The Picture condition had a significant main effect on the RT (*F* = 10.902, *p* < 0.001). Pairwise comparisons between the different Picture conditions (see Fig. 3) revealed slower responses for false beliefs than social scripts (*p* < 0.001) and mechanical stories (*p* < 0.001), and for true beliefs than social scripts (*p* < 0.005) and mechanical stories (*p* < 0.001). Following the same pattern as observed in accuracy, a significant interaction between Stimulation and Session was found (*F* = 7.968, p = 0.005), showing that participants were slower overall after stimulation compared to sham during the first session, but not during the second session.

Taken together, the behavioral results suggest that performance was worse on the critical beliefs stories compared to the social routine and non-social control stories. However, contrary to the hypotheses, performance was worse for all stories after stimulation versus sham during the first session.

### fMRI results

We hypothesized that tDCS would increase activation in social mentalizing areas of the cerebellum and cerebrum, especially for pictures requiring false and true belief mentalizing. Therefore, we focused our analysis on the contrast between tDCS and sham, conducted for each of the Picture conditions (false belief, true belief, social script, mechanical). We first discuss our analysis of the hypothesized ROIs, and then report the remaining results of an exploratory whole-brain analysis.

### ROI analysis

We performed a small volume analysis in ROIs from the mentalizing and action sequencing networks derived from earlier meta-analyses. As an exploratory analysis, we also included ROIs from the goal-directed action observation network.

For the ROI analysis we tested eight different contrasts. As seen in Table 2, only the stimulation < sham contrast revealed differences between the picture conditions, at or close to FWE-corrected significance (*p* < 0.05 or *p* < 0.10 respectively). Particularly after stimulation, the true belief condition revealed lower activity in the right TPJ, precuneus, right anterior intraparietal sulcus (aIPS) and right posterior superior temporal sulcus (pSTS). Activation was also lower in many of these regions in the social script condition (precuneus and right aIPS) and mechanical condition (precuneus and right pSTS). In these last two conditions, activation in the premotor cortex was also close to significance.

**Table 2:** *Regions of interest for social cognition and their activation in stimulus* versus *sham contrasts*.

Contrast and anatomical label		cluster-level		peak-level		peak MNI coordinates		
		p (FWE)	voxels	p (FWE)	Z	x	y	z
**True belief stimulation < sham**								
TPJ	R	0.02	90	0.02	3.43	46	−56	20
	L	0.10	10	0.07	2.96	−44	−48	22
Precuneus		0.01	190	0.04	3.19	−10	−60	40
				0.04	3.16	−8	−60	36
				0.05	3.15	2	−64	34
				0.05	3.14	8	−60	42
aIPS	R	0.06	31	0.04	3.23	40	−40	56
				0.10	2.83	40	−32	52
				0.13	2.71	36	−32	50
pSTS	R	0.08	21	0.03	3.34	46	−56	18
**Social script stimulation < sham**								
Precuneus		0.10	12	0.07	2.99	8	−62	40
Premotor cortex	L	0.10	11	0.08	2.93	−36	6	42
aIPS	R	0.10	12	0.08	2.95	32	−44	46
**Mechanical stimulation < sham**								
Precuneus		0.02	89	0.06	3.06	4	−62	38
				0.09	2.90	0	−58	34
Premotor cortex	L	0.05	39	0.04	3.18	−46	0	42
pSTS	R	0.08	22	0.10	2.81	56	−58	10
				0.10	2.81	60	−54	10

Note: Regions of interest analysis with clusters significant at *p* < 0.10 (FWE-corrected at cluster or peak level) in a small-volume FWE correction. Stimulus < sham contrasts that are not mentioned are not significant in any of the ROIs. TPJ: temporo-parietal junction, anterior IPS: anterior intraparietal sulcus, posterior STS: posterior superior temporal gyrus.

### Whole brain analysis

**Fig. 4:**
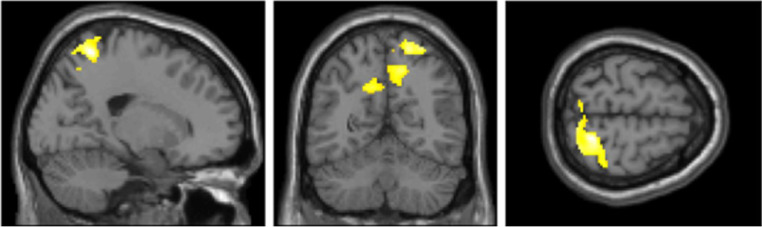
Main clusters in the primary somatosensory area (postcentral gyrus) and superior parietal lobule (precuneus) given the true belief stimulation < sham contrast from the whole-brain one-way within subjects ANOVA. MNI coordinates from left to right: *x* = 20 *y* = −59 *z* = 65.

Furthermore, we explored the same eight contrasts in a whole-brain analysis using a one-way within-participants ANOVA. We only found significant clusters in the true belief condition when contrasting stimulation < sham (Fig. 4 and Table 3). The largest cluster was in the right superior parietal lobule and postcentral gyrus. The second and third cluster included left and right insula, inferior frontal gyrus (IFG) and left caudate. A fourth cluster was located around the left primary somatosensory cortex, precentral gyrus, and superior frontal gyrus (SFG).

**Table 3:** *Whole brain analysis in stimulus* versus *sham contrasts*.

Contrast and anatomical label		cluster-level		peak-level		peak MNI coordinates		
		p (FWE)	k	p (FWE)	Z	x	y	z
**True belief stimulation < sham**								
Superior parietal lobule	R	0.00	1770	0.29	4.14	20	−54	62
Primary somatosensory				0.50	3.93	56	−18	50
				0.62	3.83	46	−28	60
Caudate	L	0.00	1473	0.37	4.04	−20	−8	24
Insula				0.49	3.94	−30	−8	16
IFG				0.67	3.79	−38	30	8
Insula	R	0.06	428	0.98	3.36	36	28	6
IFG				0.99	3.32	38	38	2
				0.99	3.13	46	30	14
Primary somatosensory	L	0.08	387	0.66	3.80	−46	−24	58
Precentral gyrus				0.71	3.76	−32	−14	68
SFG				0.96	3.34	−26	0	70

Note: Whole-brain analysis with clusters significant at *p* < 0.10 (FWE-corrected at cluster level). Stimulus < sham contrasts that are not mentioned are not significant. IFG: inferior frontal gyrus, SFG: superior frontal gyrus.

Taken together, contrary to our hypothesis, activation generally decreased after stimulation rather than increased. This pattern is in line with the decreased performance on our behavioral measures (albeit only during the first session).

## Discussion

The goal of this study was to demonstrate a causal role of the cerebellum in social sequences. Therefore, we investigated the effects of cerebellar tDCS on a picture sequencing task in which participants were asked to generate the correct order of cartoon-like stories which include false beliefs, true beliefs, social scripts and non-social mechanical events. Anodal tDCS over the right posterior cerebellum decreased behavioral performance during the first session, but not during the second session. Accordingly, tDCS decreased brain activation in cerebral regions of the mentalizing and goal-directed action observation networks, particularly during the true belief condition. No changes were observed in the cerebellum.

The negative effects of cerebellar stimulation were contrary to our hypothesis which predicted an increase in performance and brain activation. One possible explanation is that the (assumed excitatory) effect of anodal stimulation might not apply to the cerebellum due to the inhibitory output that the cerebellum has towards the cortex (Galea et al., 2009). Even though we did not observe effects on the cerebellum itself, increased activity in the cerebellum could have increased the inhibition that the cerebellum exerts over other brain regions. A previous experiment using resting state fMRI has indeed found increased inhibitory output from the right Crus I after anodal tDCS (Stoodley et al., 2017). This would explain the lower performance in the task and reduced brain activity in functionally connected regions after stimulation.

The slower response times for false and true belief events in comparison with social scripts and mechanical events, are consistent with previous literature (Heleven et al., 2019). The false and true belief conditions depict more complex situations than those shown in the social and mechanical scripts. Consequently, it is expected that accuracy would be lower and reaction times would be higher in the belief conditions indicating lower performance in these highly complex situations.

The observed interaction between session and stimulation, which revealed that participants performed worse after stimulation in the first session, but not at the second session, might have been caused by a learning effect. That is, the participants might have improved their performance in the second session so much that the weak inhibitory effects of the tDCS could only be seen in the first session. A similar learning effect has previously been observed by Heleven et al. (2021) after TMS in the picture sequencing task.

Although the picture task has previously revealed a strong activation of the cerebellum (Heleven et al., 2019), we found no effect of tDCS on the cerebellum itself. A possible explanation could come from competing effects between the anode and the cathode over the cerebellum. In this scenario, due to the spread of the tDCS current, the net effect over relatively big areas of the cerebellum would be zero, while still having an excitatory or inhibitory influence in remote cerebral areas.

Even though we did not see any significant increase or decrease of activation in the cerebellum due to tDCS, we did observe changes in activation in other areas of the mentalizing network such as the TPJ and precuneus. A previous study using dynamical causal modeling with the picture sequencing task (Van Overwalle et al., 2019a) identified closed loops between the bilateral posterior cerebellar lobes and the bilateral TPJ. Therefore, we hypothesize that the changes in activation observed in the TPJ are due to the closed-loop connections with the cerebellum. The same study provided evidence of a unidirectional connection from the cerebellum to the precuneus, which also was affected by the stimulation in this study. These results are also in line with a multi-study functional connectivity analysis (Van Overwalle & Mariën, 2016) showing that the right TPJ and the precuneus are significantly connected to the right posterior cerebellum.

This pilot study encountered several limitations. First of all, there was a relatively limited number of participants. Due to changes in the hardware and software of the scanner where the data was collected, it was not possible to increase our sample size. However, previous studies using this or similar tasks and tDCS protocols used similar sample sizes, including Heleven (2019; *n* = 24), Ballard (2019; *n* = 28), Salehinejad (2021; *n* = 16), and Oltrati (2021; *n* = 24). Since it is well-known that tDCS has differential effects in different individuals (Li et al., 2015) having a low number of participants might result in heterogeneous effects of tDCS. Consequently, even though we choose a specific montage in order to maximize focality over our target (the right posterior cerebellum), its effects may differ between participants due to an uneven distribution of the electrical field. This may have led to weaker stimulation results overall. In addition, due to time constraints when scheduling the participants, we could not keep a constant time in between sessions, which could have led to irregular learning effects among participants. On the other hand, given the presence of significant results in the present analysis, it seems promising to continue in this direction.

Future studies that wish to use the picture sequencing task in a within-participant design should explore the optimal time in between sessions to minimize learning effects. Future research could also apply different cerebellar tDCS montages (following Rice et al. 2021) to test these potential explanations.

In conclusion, although we did not find direct effects on the (posterior) cerebellum, we have provided evidence that cerebellar tDCS influences the mentalizing network. This evidence supports the hypothesis that the cerebellum has an active role in mentalizing social sequences. Advances in the field of electric current simulations will allow us to explore if the spread of the current has a direct relation with the unexpected results. Moreover, analysis of the current spread in combination with connectivity analysis could improve our understanding of the remote effects of cerebellar tDCS on the mentalizing network.

## Declaration of Competing Interest

The authors have no conflicts of interest to disclose.
